# Patent Foramen Ovale and Closure Technique with the Amplatzer Occluder

**DOI:** 10.1155/2014/129196

**Published:** 2014-06-25

**Authors:** Bernhard Meier

**Affiliations:** Cardiovascular Department, Bern University Hospital, 3010 Bern, Switzerland

## Abstract

Proof that percutaneous closure of the patent foramen ovale (PFO) is superior to medical treatment is still incomplete. Paradoxical embolism is a rare event occurring over decades rather than years. None of the 4 randomized trials published carried enough patients or was followed up for long enough to reach superiority endpoints. All data, however, point to a benefit of PFO closure. Free wall erosion (exceedingly rare) and triggering of atrial fibrillation (in about 1% of patients) are the only noteworthy complications. They are outweighed by the supposedly prevented events of paradoxical embolisms, such as stroke, transient ischemic attacks, myocardial infarctions, or other systemic embolisms. Medical treatment with perhaps the exception of lifelong oral anticoagulation provides less protection. During a 10-year follow-up of a comparative study the annual mortality was significantly lower in the patients with PFO closure (0.4%) than in those with medical treatment (1.1%, *P* < 0.03). PFO closure can be accomplished in less than 1 hour with immediate resumption of physical activity. It represents thus a kind of mechanical vaccination.

## 1. Introduction

The patent foramen ovale (PFO) has been recognized for over a century as a plausible cause of stroke permitting paradoxical embolism of a per se rather innocuous small venous clot that would go clinically unnoticed if it embolized to the lungs where it would be lysed by intrinsic fibrinogen activators in a day or two [1]. Percutaneous closure of atrial septal defects (ASDs) preceded percutaneous coronary intervention (PCI) by a couple of years when it was first described in 1974 [2]. PCI found an almost immediate resounding echo being to date considered the origin and the main representative of interventional cardiology. PFO closure only caught modest attention when it was described as a derivative of ASD closure in 1992 [3].

**Figure 1:**
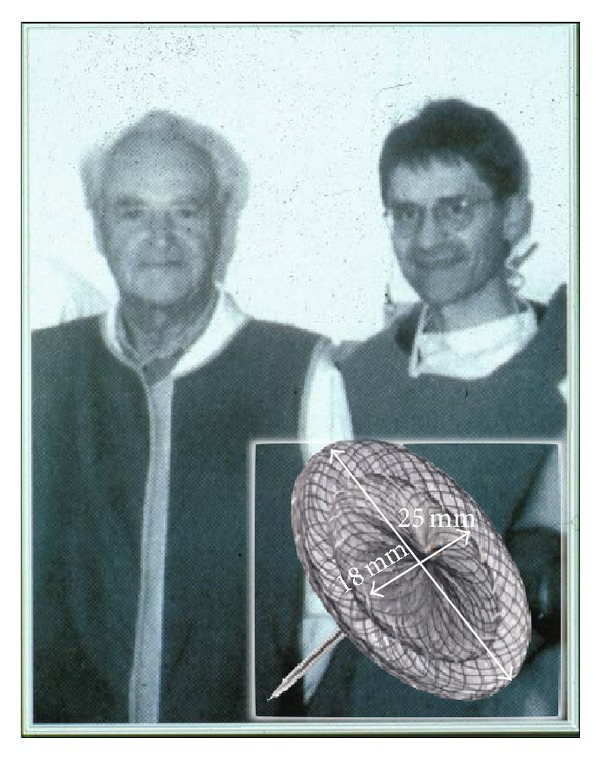
Kurt Amplatz (left) and Bernhard Meier (right) on the occasion of the world's first closure of a patent foramen ovale using an Amplatzer PFO occluder (insert) on September 10, 1997, in Switzerland.

While the initial devices were far from perfect, the clinical introduction of the Amplatzer PFO occluder (APO) in 1997 by Kurt Amplatz and the author on September 10, 1997 (Figure 1), provided a user-friendly, technically highly successful, and particularly safe method that has prevailed since without any basic changes [4]. The only truly competitive techniques are those that copied the principle of the APO.

## 2. Technique of PFO Closure

The technique developed by the inventor and the author is very straightforward and stripped to the bare essential. In particular, there is no intraprocedural echocardiography involved in contrast to the policy of most centers. While additional imaging has a potential to enhance safety and quality, it also harbors additional risks, for example, by prolonging the procedure which my engender clot-forming in the indwelling gear.

**Figure 2:**
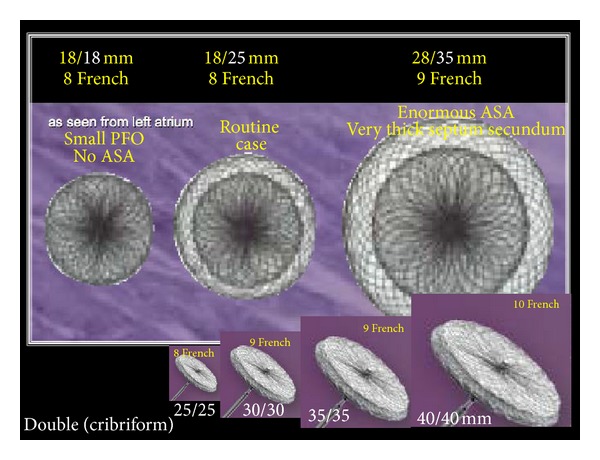
Family of Amplatzer PFO occluders. The right/left numbers indicate the diameters of the left and right disk, respectively (different in some devices). The French size indicates the minimal inner lumen of the required sheath (1F = 0.3 mm). The double (cribriform) occluders are also predestined for cribriform atrial septal defects.

All that is required is a set consisting of a TorqVue sheath with its obturator, a pusher cable, a short loader sheath, and a Y-connector with adjustable fitting. The 9 French (F) size sheath fits all APOs except for the 40/40 mm cribriform ASD occluder which is the largest member of the Amplatzer device families predestined for PFO occlusion (Figure 2). The occluder itself consists of a nitinol mesh double disk with a thin and stretchable waist with polyethylene (Dacron) inlays. While the nitinol is not absorbable and a permanent implant, the Dacron fabric inlays enhance tissue ingrowth before they are resorbed. While the original size with a 25 mm right disk and 18 mm left disk diameter (Figure 1) remained the most common, more sizes have been added (Figure 2).

The patient does not usually need sedation. A bolus of 5,000 units of heparin is given. After local anesthesia of the groin a regular 0.035 inch U-shape tip guidewire is introduced through a puncture needle. In about half of the cases the wire will readily pass through the PFO into the left atrium. If not, a curved catheter (ideally multipurpose shape) placed at the level of the diaphragm will direct the wire medially. If the PFO still cannot be passed, it is negotiated with the catheter alone and if that fails with the leading wire after straightening the U-tip. Occasionally it will be necessary to inject contrast medium to the direction where the PFO is suspected to learn more about anatomy before passing the PFO. A typical situation for that would be a PFO that consists only of a small hole in one of the corners of the initial foramen. In this case it will be necessary to guide the wire to a sharp turn once it has entered the PFO tunnel.

Next the device is prepared by screwing it loosely onto the pusher cable and retracting it in a water bath into the short loading sheath to avoid trapped air bubbles. It is wise to push it out of the loading sheath just once and retract it back while staying under water. Repeated loading maneuvers may temporarily impede the formation of flat disks.

The device is now introduced into the TorqVue sheath which was advanced into the left atrium where the obturator was slowly withdrawn allowing for back bleeding while holding the outer end of the sheath as low as possible (usually on the right side of the patient's thigh). For this, the short loading sheath is connected to the TorqVue sheath and the device is advanced close to the tip of the sheath. Air that is entering the sheath behind the device is of no concern at that point. Before exiting the sheath the safe but slightly loosened attachment of the screw is checked on fluoroscopy. If there is no gap between the screw and its female counterpart on the device, the pusher cable is turned leftward until it is felt that the apparently too tightly fit screw is now freely turning. The gap mentioned before will now appear on fluoroscopy.

**Figure 3:**
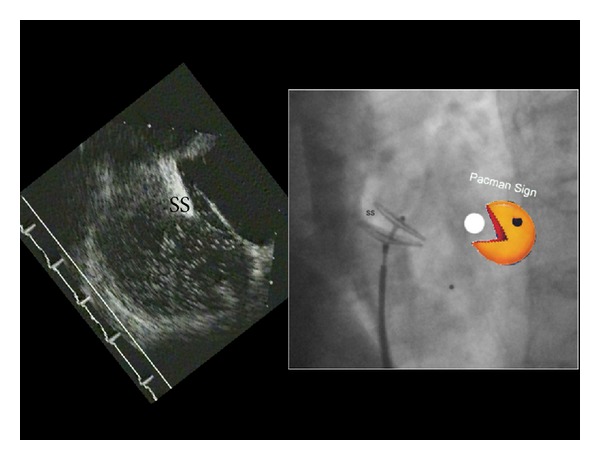
Correct placement of an Amplatzer PFO occluder ascertained by the Pacman sign. The transesophageal echocardiogram at the left is depicted in the projection usually used on fluoroscopy during implantation. The septum secundum (SS) has to be bitten into by the left more cranial parts of the occluder disks. This reminds us of Pacman biting into a dot.

The left half of the device is pushed out of the sheath up to the middle waist so that the left disk can fully form. The sheath and the pusher are then pulled back as a unit until the left disk gets stopped at the septum. In a left anterior oblique (LAO) projection the left disk will now appear in its profile. From there on only the sheath will be pulled back while gently advancing on the pusher cable. As soon as the tip of the sheath has passed the screw, the entire set is pushed against the septum to put the right disk into its proper place. At this point the typical Pacman sign should be apparent in the LAO projection (Figure 3).

The stability of the position is checked with a strong wiggle on the pusher cable and a dye injection delineating the right atrial septal border in a projection showing the 2 disks in profile and perfectly separating them (Figure 3). Then the device is unscrewed and the final position is redocumented with a right atrial dye injection after perhaps adjusting the projection to see again the 2 disks in perfect profile and completely separated.

The patient can well compress the femoral venous puncture himself or herself as there is no risk of hematoma in a supine person. The pressure in the femoral vein is 10 mmHg or less. The patient may even get up immediately but then the pressure on the groin has to be increased to about 60 mmHg because of the weight of the blood column from the top of the head to the groin.

The patient receives a tablet each of acetylsalicylic acid, 100 mg, and clopidogrel, 75 mg, to continue the former daily for 5 and the latter for 1 month. We usually perform a control transesophageal echocardiography (TEE) about 1 month after stopping all medications, that is, at about 6 months.

No restrictions are asked for and even sports are permitted immediately. Usually a transthoracic echocardiogram is performed to document the device in place before the patient leaves the hospital after an hour or so of surveillance.

Antibiotic coverage during the procedure is used in most places, for example, by giving 1 g of a cephalosporin by mouth before the procedure and another one a couple of hours later. Most centers also recommend observing the usual prophylaxis against endocarditis at least for the first 2 months.

## 3. Indications for PFO Closure

Table 1 depicts the potential indications for PFO closure referenced in the literature today and expanded by personal experience and data. Secondary prevention in the case of a recurrent presumably paradoxical embolic event on treatment with acetylsalicylic acid or oral anticoagulation is the only indication unequivocally accepted worldwide.

In light of the ease and safety of PFO closure and the compelling evidence of its efficacy in the prevention of several types of events, it appears mandatory to react to the first event already. Even in case that the event was not related to the PFO, the fact that the PFO was closed without any untoward effects (almost invariably the case) prevents the subject from any of the PFO-mediated problems in Table 1 for good, resulting in a net benefit.

The indication to close a PFO should not be subject to the absence of other potential stroke causes or stroke facilitators such as the ones listed in Table 2. To the contrary, PFO closure should be the first thing to consider with any stroke as it is one of the easiest causes for stroke to assess and the easiest to remedy.

Prothrombotic predisposition is common in the general population and should be a reason to close the PFO rather than not to. Even if the predisposition is considered severe enough to warrant lifelong oral anticoagulation, the thrombotic risk with the possibility of paradoxical embolism should still be considered high enough to warrant PFO closure.

## 4. Randomized Evidence for the Effect of PFO Closure

A total of 4 randomized PFO closure trials have been published so far. The first one pertained to the role of the PFO in migraine and is called MIST trial (Migraine Intervention with STARFlex Technology) [5]. In this European sham-controlled single-blind randomized trial, patients with a documented PFO and frequent migraine attacks not controlled with 2 or more classes of prophylactic drugs were screened and enrolled by headache specialists to either have their PFO closed or to undergo general anesthesia and receive a skin nick in the groin without having a device implanted (sham-control). The obsolete STARFlex device was used, one of the devices with the poorest records among those that have been used for PFO closure during the past 20 years. The follow-up duration was 6 months. Of a total of 432 patients screened, 260 (60%) were found to have a PFO. This is clearly higher than the prevalence of PFOs in the normal population which could be explained by a common genetic background were it not for the therapeutic effect of PFO closure evident in this trial (albeit not to a significant degree) and in most other respective nonrandomized reports. Women accounted for 85% of the patients and the average age was 45 years. Roughly 40% in the PFO closure group benefitted from a reduction of headache days of at least 50% compared to roughly 20% in the sham-control group. The* P* value was borderline. Unfortunately the outcome of the MIST trial has since been more often used as a reason not to close a PFO in the realm of migraine while it should have been used to close it. First, there was a clear trend for migraine improvement and, second, the collateral benefit of protection against other more important events should be taken into account. For instance, 1 of the 74 patients in the sham-control group suffered a stroke during the 6-month follow-up. This points to the main reason why PFOs are to be closed.

The CLOSURE I trial (Evaluation of the STARFlex Septal Closure System in Patients with a Stroke and/or Transient Ischemic Attack due to Presumed Paradoxical Embolism through a Patent Foramen Ovale) in North America was the first randomized trial to be published on PFO closure for prevention of ischemic cerebral events [6]. This randomized open-label superiority trial was hampered by a short follow-up (maximum of 2 years) and the use of the deficient STARFlex device. A total of 447 patients were randomized to closure and 462 patients to medical therapy, the kind of which was left to the discretion of the physicians. The average age was 46 years and the genders were balanced; as they were in all respective trials. The predefined primary endpoint initially defined as a reduction of stroke or transient ischemic attacks (TIAs) from 6% to 3% over 2 years was changed in-flight in light of the slow recruitment observed from 6% to 2% to allow for a reduction to 800 patients. The observed primary endpoint rates were 7.7% in the medical group and 5.8% in the closure group and this was not statistically significant. In addition, atrial fibrillation (AF) was observed in 6% in the closure group versus 1% in the medical group. The majority of these AF episodes, however, occurred in the first 2 weeks and were clinically silent. None of the subgroup analyses revealed a significant protection of PFO closure but none hinted to inferiority of it, either. Presumably due to the selection process and the poor performance of the STARFlex device, the closure group in the CLOSURE trial performed worse than the average performance of nonrandomized comparative trials of PFO closure and the control group performed better [7]. This explained the narrow difference.

The PC trial (Percutaneous Closure of Patent Foramen Ovale in Cryptogenic Embolism) was the next we published and had been the first to be started, about 12 years before publication by the author and his group [8]. According to what had been stated in the protocol, only the first 5 years of follow-up were analyzed. A total of 414 patients at an average age of 45 years were randomized in countries dispersed around the world to either closure of the PFO with an APO or medical therapy with antiplatelet drugs or oral anticoagulation as per discretion of the treating physician. The projected primary endpoint of a reduction of the annual recurrence rate of stroke, TIA, or peripheral embolism from 3% to 1% was not reached. However, there was a relative stroke reduction of 80% (5 strokes in the control group and 1 stroke in the closure group) which in itself leaves no doubt that medical therapy could at best be noninferior. Using the stroke definition of the similar RESPECT trial (Randomized Evaluation of Recurrent Stroke Comparing PFO Closure to Established Current Standard of Care Treatment) [9], the stroke rate was even 7-fold higher without a PFO closure. Complications were extremely rare. AF was numerically but not significantly more common in the closure group which is explicable on the basis of mechanical irritation by the device.

The RESPECT trial [9] randomized a total of 980 patients in the United States of America to either closure of the PFO with an APO or medical therapy with antiplatelet drugs or oral anticoagulation at the discretion of the treating physicians. Per protocol the follow-up was terminated when 25 strokes had occurred. By intention to treat analysis, 16 strokes had happened in the medical group and 9 in the closure group with a* P* value of 0.08. Analyzed as treated, the difference was significant (*P* = 0.007) with 16 strokes in the control group and 5 in the closure group. Moreover, predefined subanalyses showed a significant stroke reduction in patients with a substantial PFO size or an atrial septal aneurysm and when only the control patients treated with antiplatelet agents were considered. Overall, the numbers needed to treat to prevent a stroke were 70 and 24 at 2 and 5 years, respectively. Again, there was a numerically more common occurrence of AF in the closure group without statistical or clinical significance.

Lumping and meta-analyzing these 3 trials, the trend to improved outcome with PFO closure compared to medical therapy becomes stronger [10]. It reaches significance in some meta-analyses [11]. Looking only at the APO studies, significant improvement is documented [12].

There are 3 similar randomized trials still ongoing with a projected total cohort of roughly 1,700 patients, that is, the CLOSE trial in France (Closure of Patent Foramen Ovale or Anticoagulants versus Antiplatelet Therapy to Prevent Stroke Recurrence, NCT00562289), the DEFENSE PFO trial in South Korea (Device Closure Versus Medical Therapy for Secondary Prevention in Cryptogenic Stroke Patients with High Risk Patent Foramen Ovale, NCT01550588), and the REDUCE trial in Canada and Denmark (GORE HELEX Septal Occluder and Antiplatelet Medical Management for Reduction of Recurrent Stroke or Imaging-Confirmed TIA in Patients with Patent Foramen Ovale, NCT00738894). However, it is unlikely that any of these trials will be, in itself, significant for the reasons of patient numbers and follow-up duration cited above. They will add to the already sizable amount of data collected. It is close to certain that they will point to the same direction, that is, the advantage of PFO closure. The question even arises whether further randomization of patients with an index event suggesting paradoxical embolism and a proved PFO is ethical still. Recurrent events are more likely to occur in the control group and they may encompass death, debilitating stroke, or myocardial infarction and therefore be difficult to defend considering the fact that they could have been prevented by a simple procedure.

## 5. Nonrandomized, Personally Collected Evidence for the Effect of PFO Closure

**Figure 4:**
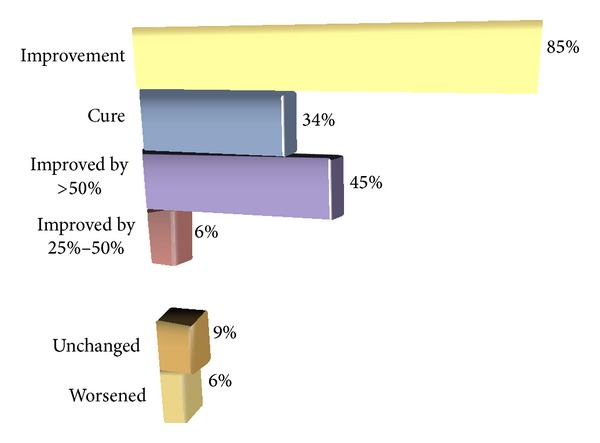
Migraine status in 150 consecutive patients with Amplatzer PFO closure at an average of 5 ± 2 years of follow-up. The average age of the patients was 51 ± 11 years, 61% were males, and 91% had complete closure by 6-month transesophageal echocardiography. Antiplatelet treatment had been stopped at the latest 6 months after the intervention.

In terms of subjective improvement, 603 consecutive patients with APO PFO closure mainly for reason of secondary prevention after neurological events were assessed as per their migraine status before and long term after PFO closure [13]. Of note, there were no acute or follow-up complications during an average of 5 ± 2 years. One patient suffered a transient ischemic attack at 4 years, accounting for a 0.15% event rate per year.  Figure 4 depicts that migraine improved dramatically. The long observation period excludes a mere placebo effect.

**Figure 5:**
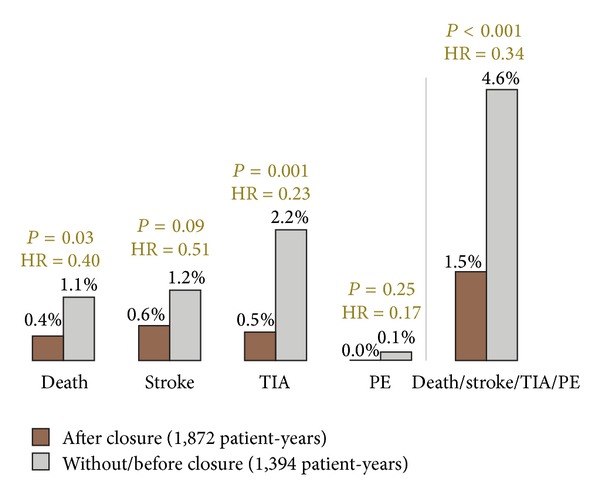
Annual event rates during a 10-year follow-up in 308 consecutive patients randomly assigned to PFO closure or medical treatment. PE = peripheral embolism and TI = transient ischemic attack.

The most compelling data recommending PFO closure originate from the author's group [14]. In the late nineties 308 consecutive patients with an index neurological event and a PFO were randomly either sent for PFO closure or kept on neurological outpatient treatment with either antiplatelets or oral anticoagulation. A median of 10 years of follow-up was available, looking at the total of 3,266 patient-years. A statistically significant mortality reduction by PFO closure was proved (0.4 versus 1.1% per year, *P* = 0.03) when the time after PFO closure was compared to that before or without closure. This was driven by a roughly 50% reduction in stroke rate (0.6% versus 1.2% per year, *P* = 0.09) and an even more drastically reduced rate of transient ischemic attacks (Figure 5).

## 6. Outlook

Uncontestably, the PFO is a blemish with a small but definite threat to health and even survival. As it is present in about 25% of the population, every PFO cannot be closed. Even if the logistics were created to screen for a PFO and close every PFO detected it might not be economically feasible. Although a prevented stroke may save sufficient money to pay for hundreds of PFO closures, some PFOs may have a lifetime risk of hardly a percent to cause significant damage. Moreover, currently reliable screening for PFO is only possible with TEE combined with a Valsalva maneuver and an intravenous injection of echocardiographic contrast medium (bubble test). If the targeted sensitivity is reduced to large PFOs, such as those with an atrial septal aneurysm, opening the gap with every heartbeat, or those with an Eustachian valve directing the inflow from the inferior vena cava directly on to the PFO, transthoracic echocardiography with a bubble test suffices. Transcranial Doppler techniques to demonstrate right-to-left shunts have been perfected over the past 20 years but they also require an intravenous injection of some media with a gaseous content. They are able to detect even small shunts but they cannot define where the shunt occurs. The same holds true for the most simple of all techniques, the screening for ear lobe oxygen dip after a sustained Valsalva maneuver without using any injection [15]. This is the only test that could be used for screening school classes. Yet its reliability, sensitivity, and specificity need to be tested further.

The PFO needs not to be looked for in childhood as venous clots are virtually inexistent at very young age. Clots tend to come into existence at middle age (with few exceptions) after which their incidence rises geometrically [16]. The common perception that the PFO is of less importance in older and sick people has to be put into perspective. The absolute PFO potential to cause harm steeply increases with age (more clots in the venous blood) but the competitive causes of ischemic events become more and more numerous so that the PFO's relative risk decreases. Some argue that the PFO will shunt less frequently with age due to the increasing left atrial pressure. However, Valsalva maneuvers during defecation and micturition (men) also intensify with age. Thus, closing the PFO makes sense even at high age and with other stroke sources present. This concept has yet to be adopted by medical attitudes guidelines and textbooks along the modified stroke classification in Table 2.

PFO closure is the most straightforward, easy, and perhaps even the most beneficial (net benefit) intervention in today's cardiology. It has to be in the repertory of every interventional cardiologist. Health authorities should not only ascertain reimbursement but also foster PFO closure. There is still a long way to go to fill the gaps in secondary prevention, not to mention exploiting the potential of PFO closure in primary prevention in terms of a mechanical vaccination.

## Figures and Tables

**Table 1:** Potential indications for PFO closure.

(i) Secondary prevention	
(a) Stroke	
(b) Transient ischemic attack	
(c) Embolic myocardial infarction	
(d) Peripheral embolism	
(e) Decompression incident	
(f) High altitude pulmonary edema	
(ii) Primary prevention	
(a) Aggravating PFO attributes	
(1) Atrial septal aneurysm	
(2) Eustachian valve	
(3) Chiari network	
(b) Prothrombotic state	
(c) Deep vein thrombosis	
(d) Pulmonary embolism	
(e) Pacemaker/defibrillator electrodes	
(f) Embolism-prone surgery	
(1) Major orthopedic	
(2) Cerebral in sitting position	
(g) Planned pregnancy	
(h) Carcinoid tumor	
(i) Special congenital situations	
(iii) Therapeutic	
(a) Migraine (with aura)	
(b) Platypnea orthodeoxia	
(c) Provoked exercise desaturation	
(d) Sleep apnea	
(iv) Vocational or recreational	
(a) Deep sea diver	
(b) Mountain climber, highlander	
(c) Brass musician	
(d) Glass blower	
(e) Tile setter	
(f) Military jet	
(g) Astronaut or commercial pilot driver	
(h) Acrobat pilot	

**Table 2:** Ischemic stroke classification.

(i) Arterial occlusion	
(a) Lacunar	
(b) Intracerebral	
(c) Vertebral	
(d) Internal carotid	
(e) Common carotid	
(f) Brachiocephalic	
(ii) Arterial embolus	
(a) Plaque/ulcer/dissection	
(1) Intracerebral	
(2) Carotid	
(3) Vertebral	
(4) Brachiocephalic	
(5) Ascending aortic	
(iii) Cardiac embolus from	
(a) Left ventricle	
(b) Left atrium	
(1) Left atrial appendage (atrial fibrillation)	
(2) Left atrial foramen pouch	
(c) Myxoma or other tumors	
(d) Vegetation (septic embolus)	
(iv) Paradoxical embolus	
(a) Patent foramen ovale	
(b) Atrial septal defect	
(c) Pulmonary fistula	
(v) Pulmonary venous bed embolus	
(vi) Cryptogenic	
